# Digital Skincare Marketing, Self-Experimentation, and Treatment Adherence in Acne Vulgaris: A Psychodermatological Review

**DOI:** 10.7759/cureus.117365

**Published:** 2026-10-02

**Authors:** Nandika Bansal, Rena Ahuja

**Affiliations:** 1 Biology, Sage Hill School, Newport Coast, USA; 2 Internal Medicine, Hoag Hospital Newport Beach, Newport Beach, USA

**Keywords:** acne vulgaris, dermocosmetics, digital skincare marketing, medication adherence, psychodermatology, skin barrier dysfunction, social media, treatment non-adherence

## Abstract

Acne vulgaris requires sustained treatment, yet adherence may weaken before meaningful improvement occurs, particularly when topical therapy causes xerosis, peeling, erythema, or stinging. Digital acne content can expand access to education and patient perspectives, but it also places clinical advice beside commercial claims, transformation narratives, and complex skincare routines without a consistent hierarchy of evidence. This narrative review examined evidence relevant to how psychodermatological vulnerability and digital skincare exposure may contribute to cosmetic self-experimentation, cutaneous irritation, and treatment non-adherence, while distinguishing observed associations from untested mechanistic links. This digital exposure includes exposure to online acne/skincare recommendations, social media advice, influencer content, transformation narratives, and algorithmically reinforced skincare-routine content. PubMed, Embase, and Google Scholar were searched through July 22, 2026, for English-language literature published since 2016. Across the reviewed literature, acne-related stigma, depressive symptoms, body dysmorphic symptoms, and treatment expectations were associated with psychological burden, treatment satisfaction, and intention to adhere. Separate studies also described patient uptake of online acne recommendations, clinician-observed dermatoses associated with multi-step cosmetic routines, and associations between cutaneous adverse effects and poorer medication adherence. Together, these findings support a conceptual model in which psychodermatological distress, delayed improvement, online product exposure, cutaneous symptoms, and adherence difficulties may interact, but the available evidence does not establish this sequence causally or longitudinally. No longitudinal study measured digital exposure, product additions, objective barrier change, symptom attribution, and medication persistence in the same patients. Acne care should therefore emphasize realistic treatment timelines, application demonstration, regimen simplification, early follow-up, and detailed review of prescribed and non-prescribed products. These findings do not justify treating all digital content or non-prescription skincare as harmful. Clinician-produced content may support patient education, and selected dermocosmetics may have a role as adjuncts to structured acne care.

## Introduction and background

Acne vulgaris is a chronic inflammatory disorder of the pilosebaceous unit and one of the most prevalent skin diseases globally, affecting 9.4% of the global population in 2010 and ranking as the eighth most prevalent disease worldwide [1]. Evidence-based care is guided by lesion morphology, severity, scarring risk, tolerability, patient preference, and psychosocial burden, with strong clinical recommendations supporting benzoyl peroxide, topical retinoids, topical antibiotics, oral doxycycline, and oral isotretinoin for severe, scarring, treatment-resistant, or psychosocially burdensome disease [1]. This therapeutic standard places acne treatment within a longitudinal disease-control model: reduction in inflammatory and noninflammatory lesion count, prevention of post-inflammatory dyspigmentation and scarring, and maintenance of remission through sustained exposure to appropriate pharmacologic agents. It is not a cosmetic trial-and-error process. Effective acne care requires time.

Therapeutic adherence remains fragile during the earliest phase of treatment. Acne management involves numerous topical and systemic options, long-term disease control, and active patient cooperation, yet undertreatment and non-adherence persist despite available therapies [2]. The first failure point occurs before many regimens have had adequate biological time to work. Topical treatments are first-line for mild-to-moderate acne, but persistence is limited when patients experience xerosis, peeling, erythema, stinging, or irritation without immediate visible reduction in lesion burden. Counseling must account for the expected six-to-eight-week interval before meaningful improvement is typically observed; without that anticipatory guidance, early adverse effects can be misread as therapeutic failure [3]. The adherence problem begins inside this mismatch between pharmacologic time and cosmetic urgency.

Treatment friction may occur within a measurable psychosocial context. Acne carries psychodermatological consequences that extend beyond visible lesions, with appearance comparison, stigma, avoidance behavior, and media-shaped beauty ideals contributing to patient distress [4]. Quantitative data from a cross-sectional cohort of 150 patients with acne vulgaris link acne severity with depression, anxiety, and stress, while regression modeling demonstrates negative effects of acne and psychological distress on self-esteem and quality of life [5]. The patient waiting for lesion resolution may be managing embarrassment, social vigilance, diminished confidence, and a need to regain control over a highly visible disease. The behavioral relevance of digital exposure is already observable: among 358 adult female acne patients, 20.9% reported willingness to alter physician-prescribed treatment based on social media advice [6].

This review examines how psychodermatological vulnerability and digital skincare marketing interact to shape cosmetic self-experimentation, cutaneous irritation, and treatment adherence in acne vulgaris, and evaluates which clinician-led responses are supported by the current evidence. The literature does not establish a single longitudinal causal chain. It does, however, support a hypothesized conceptual pathway: appearance-related distress may narrow tolerance for delayed improvement; digital content can supply highly visible product-centered alternatives; multi-product exposure may increase irritant and allergen burden; and treatment-associated or product-induced symptoms may contribute to premature discontinuation (Figure 1).

**Figure 1:**
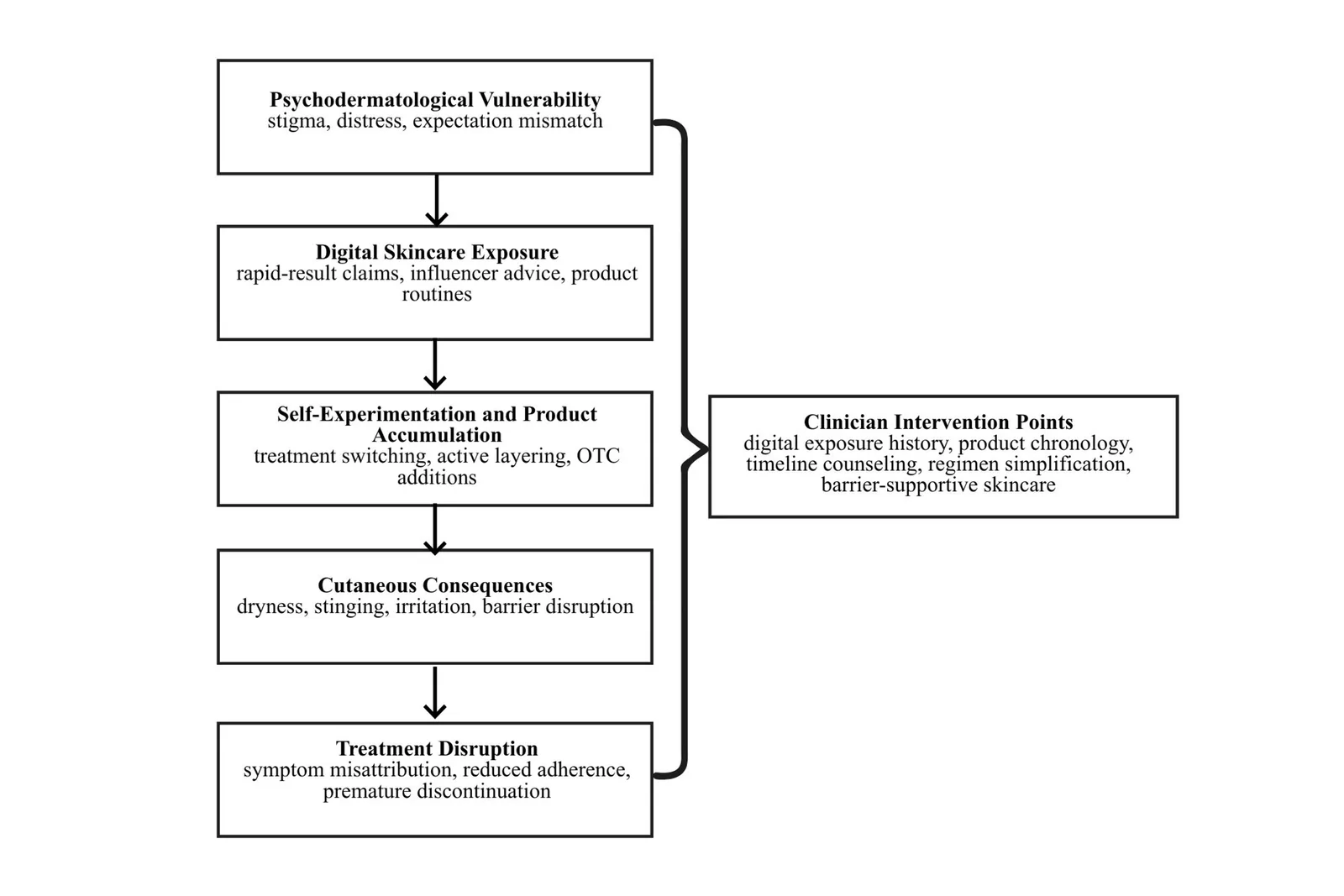
Hypothesized conceptual model linking digital skincare marketing with acne treatment disruption The model summarizes proposed relationships among psychodermatological vulnerability, digital skincare exposure, product self-experimentation, cutaneous symptoms, and adherence disruption. It is intended to organize observed associations and identify clinician intervention points, not to imply that the full sequence has been longitudinally demonstrated.

The sections that follow evaluate the evidence for each stage separately, identify where independent findings converge, and distinguish established associations from mechanisms that require prospective testing.

## Review

Literature search strategy

A targeted literature search was conducted between June 24 and July 22, 2026, across PubMed, Embase, and Google Scholar to identify English-language evidence published from 2016 through July 22, 2026. Database-specific controlled vocabulary and free-text terms were adapted for each database.

Searches used combinations of the following concepts: (“acne vulgaris” OR acne) AND (“medication adherence” OR adherence OR non-adherence OR nonadherence OR persistence OR “patient compliance”); (“acne vulgaris” OR acne) AND (“social media” OR TikTok OR Instagram OR YouTube OR internet OR “online information” OR influencer* OR misinformation); (“acne vulgaris” OR acne) AND (psychodermatology OR stigma OR depression OR anxiety OR “body dysmorphic disorder” OR “quality of life” OR “self-esteem” OR “treatment satisfaction”); (“acne vulgaris” OR acne) AND (skincare OR “skin care” OR cosmeceutical* OR dermocosmetic* OR “skin barrier” OR irritation OR xerosis OR stinging OR dermatitis OR “contact allergen”); and (skincare OR “skin care” OR cosmeceutical* OR dermocosmetic*) AND (“social media” OR influencer* OR “electronic word-of-mouth” OR “source credibility” OR “purchase intention”). Google Scholar was used as a supplementary source to identify relevant digital media, consumer-behavior, and dermatology literature not retrieved in the database searches.

Records were screened in a stepwise manner: duplicate or clearly irrelevant records were removed, titles and abstracts were screened for relevance to acne management and adherence, psychodermatological vulnerability, digital content quality, consumer-product exposure, or clinician response, and full texts were reviewed for direct relevance to treatment adherence, digital product use, skin barrier injury, cutaneous adverse effects, or clinician-led adherence interventions. Reference lists of selected publications were manually searched for additional evidence. Included patient, clinical, and consumer-behavior studies represented multiple international settings, including Saudi Arabia, Thailand, Turkey, the Netherlands, and Sri Lanka.

Non-English literature was excluded because formal translation was not available. Single-patient case reports and small case series were excluded because this review focused on population-level behavior, digital content environments, clinician-observed trends, and adherence patterns rather than rare individual adverse events. Individual social media posts, brand websites, industry white papers, promotional skincare materials, and non-peer-reviewed marketing reports were not used as principal evidence. Regulatory documents were not used as principal sources because the review focused on patient behavior, product exposure, and treatment adherence rather than legal classification of over-the-counter products.

This review was conducted as a narrative synthesis rather than a registered systematic or scoping review because the research question integrated heterogeneous evidence from psychodermatology, digital media analysis, consumer marketing, dermatologic adverse effects, and medication adherence. The aim was to synthesize converging mechanisms and practice implications rather than estimate pooled effect sizes or answer a single intervention-outcome question. Formal duplicate screening, quantitative meta-analysis, and risk-of-bias scoring were not performed. To reduce interpretive bias, eligibility categories were defined before final synthesis, reference lists were manually searched, borderline inclusions were rechecked against the review question, and principal sources were evaluated according to study design, sample size, directness to acne treatment behavior, and whether exposure and outcome were measured within the same population.

The principal sources directly informing this conceptual model are summarized in Table 1. 

**Table 1:** Principal sources and evidence domains informing the proposed conceptual model Sources were grouped according to the primary evidence domain they informed in the narrative synthesis: acne management and adherence, psychodermatological vulnerability, digital content quality, consumer-product exposure, and clinician response. These domains are not mutually exclusive; several sources inform more than one part of the proposed model. Each source is summarized according to its design or material, principal finding or argument, relevance to the review question, and main interpretive constraint. DISCERN, instrument for assessing the quality of consumer health information; Global Quality Scale, measure of overall information quality and clinical usefulness.

Source	Primary evidence domain	Design and sample or material	Principal finding or argument	Relevance to the review question	Main interpretive constraint
Morshed et al., 2023 [5]	Psychodermatological vulnerability	Cross-sectional study; 150 patients with clinically diagnosed acne vulgaris	Acne severity and psychological distress were associated with lower self-esteem and poorer quality of life.	Defines the psychodermatological burden within which patients evaluate treatment progress.	Digital exposure, product use, and adherence were not measured; temporal direction cannot be determined.
Ertekin et al., 2024 [6]	Consumer-product exposure	Cross-sectional survey; 358 adult female acne patients	Among participants seeking acne information online, 88.4% followed at least one recommendation, 20.9% reported willingness to alter prescribed treatment, 12.8% reported worsening, and 0.8% reported significant improvement.	Provides direct evidence that online recommendations enter acne-related treatment behavior.	Adult female, self-reported sample; specific exposures, objective cutaneous outcomes, and symptom attribution were not measured.
Tunca et al., 2025 [7]	Psychodermatological vulnerability	Cross-sectional study; 210 patients aged 14-40 years	Acne severity correlated modestly with quality-of-life impairment and stigma, whereas stigma correlated more strongly with depression, social anxiety, and quality-of-life impairment.	Shows that perceived stigma may be more closely related to psychological morbidity than lesion severity alone.	Cross-sectional design prevents determination of causal or temporal direction.
Tros et al., 2023 [8]	Psychodermatological vulnerability	Two-center cross-sectional study; 105 dermatology patients aged 13-24 years	Approximately one in eight screened positive for body dysmorphic disorder, and affected patients had lower self-esteem.	Identifies a subgroup in whom perceived defect and objective lesion burden may diverge.	Screening did not establish a psychiatric diagnosis; the small specialty-care sample limits generalizability, and adherence was not assessed.
Alghofaili et al., 2021 [9]	Psychodermatological vulnerability	Cross-sectional study; 204 Saudi acne patients	Depression was negatively associated with treatment satisfaction (β=-0.121), expected medication effect was positively associated with satisfaction (β=0.334), and satisfaction was positively associated with intention to adhere (β=0.1713).	Links emotional state and treatment expectations with satisfaction and adherence intention.	The study measured intention rather than observed medication use, and its cross-sectional design prevents causal inference.
Thang et al., 2023 [10]	Digital content quality	Cross-platform content analysis; 250 acne-treatment videos from TikTok, Instagram Reels, and YouTube Shorts	Dermatologist-produced videos had higher DISCERN and Global Quality Scale scores, while layperson posts had significantly higher engagement than dermatologist posts.	Documents a mismatch between clinical quality and audience engagement.	The analysis captures a cross-platform snapshot at one point and did not measure individual exposure or treatment behavior.
Irfan et al., 2023 [11]	Digital content quality	Cross-sectional content analysis; 100 most-liked #acne TikTok videos	Thirty-eight videos were physician-created, and DISCERN scores remained low across creator categories.	Shows that highly visible acne content may remain limited in evidentiary quality regardless of creator type.	Popularity-based sampling and changing platform content limit generalizability; viewer outcomes were not measured.
Iyengar et al., 2025 [12]	Digital content quality	Thematic qualitative analysis; 150 leading #acne TikTok videos	Medically trained clinicians produced 11% of content; education appeared alongside pimple-popping, transformation narratives, acne positivity, and “acne reality” content.	Defines the mixed informational and emotional environment in which acne claims are encountered.	Platform-specific content analysis cannot determine how viewers interpret or act on the material.
O’Connor et al., 2022 [13]	Digital content quality	Qualitative review of acne misinformation and conspiracy narratives	Recurring themes included exaggerated dietary causation, unconventional cures, distrust of conventional treatment, and product-affiliated rapid-result claims.	Identifies messages that may alter therapeutic expectations and encourage treatment substitution.	The review characterized misinformation rather than measuring patient exposure, prevalence, or treatment behavior.
Fomiatti, 2026 [14]	Consumer-product exposure	Conceptual media analysis of cosmeceutical advertising	Scientific vocabulary and expert-coded claims were described as forms of “epistemic consumption” that make cosmetic purchasing appear medically informed.	Supplies a plausible mechanism through which product marketing can resemble clinical reasoning.	The analysis was not acne-specific and did not measure purchasing, regimen complexity, or adherence.
Stefana and Damiani, 2026 [15]	Consumer-product exposure	Dermatologic commentary	“Cosmeticorexia” was proposed as descriptive language for excessive or age-inappropriate cosmetic use.	Provides terminology for the product-overuse behavior considered in the review.	The article presents no original data, validated diagnostic criteria, or prevalence estimate.
Hales et al., 2025 [16]	Consumer-product exposure	Content analysis of 100 videos plus experimental engagement through two accounts registered as 13-year-old users	Routines averaged six products and $168; 26.2% of daytime routines included sunscreen, and 76% of the most-viewed routines contained a potential contact allergen. Engagement increased subsequent exposure to similar content.	Quantifies the complexity and ingredient burden of modeled routines and documents algorithmic reinforcement after engagement.	The content involved pediatric skincare generally rather than acne treatment; viewer adoption and clinical outcomes were not measured.
Poelhekken and van der Bent, 2026 [17]	Consumer-product exposure	National survey; 168 Dutch dermatologists and dermatology residents	Among respondents, 91.6% expressed concern about multi-step routines and 88.1% regularly encountered skincare-related dermatoses.	Supplies clinician-observed evidence of the complication stage proposed in the pathway.	Exposure histories, social-media origins, and patient-level causation were not verified.
Aleissa et al., 2026 [18]	Acne management and adherence	Cross-sectional study; 198 patients with dermatologist-confirmed acne	Non-adherence was 50.5% and was associated with adverse effects, particularly dryness and stinging, as well as topical therapy and moderate severity.	Links cutaneous adverse effects with non-adherence.	Digital exposure and non-prescribed skincare use were not measured, so the source of adverse effects cannot be determined.
Ferhatosmanoğlu et al., 2026 [19]	Acne management and adherence	Multicenter study; 2,349 patients across 18 Turkish dermatology centers	Forty-eight percent reported consistent medication use; barriers included forgetfulness, dislike of treatment, and reluctance toward long-term therapy.	Identifies duration, convenience, and treatment acceptability as recurring adherence frictions.	Adherence was self-reported, and digital exposure was not assessed.
Paichitrojjana and Paichitrojjana, 2025 [20]	Acne management and adherence	Cross-sectional analysis; 1,237 Thai participants	Adherence was 33.71%; cost, adverse effects, and lack of noticeable improvement were major barriers, while convenience and aesthetics influenced treatment selection.	Shows that therapeutic and consumer-oriented considerations coexist in real-world acne decisions.	Country-specific, self-reported data; digital influence was not directly measured.
Bal et al., 2025 [21]	Clinician response	Cross-sectional survey; 100 acne patients	Ninety-seven percent favored dermatologist guidance when it conflicted with influencer advice, yet 67% continued to seek acne information from influencers.	Shows that professional trust and continued online information seeking can coexist.	Small clinical sample; information seeking was measured rather than purchases, regimen changes, or adherence.
Ranjith et al., 2025 [22]	Consumer-product exposure	Cross-sectional survey with structural equation modeling; 384 Sri Lankan Facebook skincare purchasers	Source credibility, electronic word-of-mouth credibility, and community trust were positively associated with purchase intention; perceived risk was negatively associated.	Provides consumer-behavior evidence for how credible-seeming online information may influence skincare product evaluation.	The study was not acne-specific and measured purchase intention rather than actual purchasing or prescription adherence.
Moradi Tuchayi et al., 2016 [23]	Clinician response	Narrative review of interventions to increase acne treatment adherence	Reported adherence barriers include regimen complexity, adverse effects, weak communication, forgetfulness, and lack of perceived efficacy; reviewed interventions include counseling, application demonstration, regimen simplification, and follow-up.	Supports practical clinician-led strategies to reduce non-adherence during the early acne-treatment window.	The evidence base for acne adherence interventions is limited and heterogeneous.
Thiboutot et al., 2025 [24]	Clinician response	International expert consensus on dermocosmetics in acne	Acne-appropriate cleansers, moisturizers, sunscreens, and selected dermocosmetics may support barrier function, reduce treatment-related irritation, and improve tolerability when assigned a defined role.	Supports distinguishing structured barrier-supportive skincare from unsupervised product accumulation.	Consensus recommendations rely on variable evidence quality and expert interpretation.

Psychodermatological vulnerability and treatment expectations

Psychosocial burden is consistent across independent acne cohorts, but its components are not interchangeable. In a cohort of 150 patients, acne severity and psychological distress were associated with reduced self-esteem and poorer quality of life [5]. In a separate cohort of 210 patients aged 14 to 40 years, global acne severity correlated modestly with dermatology-specific quality-of-life impairment (r=0.196, p=0.004) and stigmatization (r=0.156, p=0.024), whereas stigmatization showed stronger associations with depression (r=0.461, p<0.001), social anxiety (r=0.336, p<0.001), and quality-of-life impairment (r=0.472, p<0.001) [7]. The difference in association strength matters for patient care: objective severity was only modestly related to stigma, while perceived stigma was more closely related to psychological morbidity and impaired daily functioning. Because both studies were cross-sectional, the direction of these relationships remains uncertain, but their convergence shows that treatment decisions occur within a measurable psychosocial burden rather than neutral consumer conditions.

Vulnerability also varies across patients. Among adolescents and young adults receiving dermatologic care for acne vulgaris, approximately one in eight screened positive for body dysmorphic disorder and showed lower self-esteem than patients without the disorder [8]. In a separate sample of 204 Saudi patients, depressive symptoms were negatively associated with treatment satisfaction (β=-0.121, p=0.033), expected medication effect was positively associated with satisfaction (β=0.334, p<0.001), and satisfaction was positively associated with intention to adhere (β=0.1713; 95% CI, 0.068-0.274) [9]. These studies identify distinct but related processes. Dysmorphic symptoms may separate perceived defect from objective lesion severity, while expectation-response mismatch is associated with lower satisfaction and weaker intention to adhere. Neither study established that dysmorphic symptoms or depression cause non-adherence.

The practical synthesis is therefore not a single vulnerable-patient profile. Stigma, dysmorphic symptoms, depressive symptoms, and treatment expectations affect different parts of how disease burden and therapeutic progress are judged [5,7-9]. These findings do not show that psychologically distressed patients go on to adopt online routines. Instead, these findings identify psychological and expectation-related factors associated with how patients may judge disease burden and treatment progress; whether these factors increase later digital self-experimentation or non-adherence remains a hypothesis requiring prospective testing.

Digital content quality, commercial persuasion, and modeled skincare routines

Short-form acne media separate evidentiary quality from visibility. In a cross-platform analysis of TikTok, Instagram Reels, and YouTube Shorts, dermatologist- and clinic-produced videos scored more highly on DISCERN and Global Quality Scale measures, whereas layperson posts generated significantly greater engagement than posts by dermatologists [10]. On TikTok, only 38 of the 100 most-liked #acne videos were physician-created; 29 were produced by non-physicians and 27 by private companies, while DISCERN scores remained low across creator categories [11]. A separate thematic analysis of 150 leading #acne videos found that medically trained clinicians produced only 11% of the content; acne education appeared alongside pimple-popping, transformation narratives, acne positivity, and unfiltered “acne reality” footage [12]. Qualitative misinformation research identified exaggerated dietary causation, unconventional cures, distrust of conventional therapies, and product-affiliated claims of rapid resolution after standard treatment failure [13].

None of these studies claimed that inaccurate content reliably outperformed accurate content, or that viewers acted on every claim they saw. The problem they revealed was subtler: creator authority, information quality, emotional salience, commercial affiliation, and audience engagement were not reliably aligned [10-13]. Clinical advice, personal testimony, transformation imagery, product advertising, and treatment distrust can therefore appear within the same feed without an evidentiary hierarchy visible to the patient. This mixed environment does not negate the educational value of clinician-produced digital content; rather, it makes source identification, evidence quality, and commercial transparency relevant.

Commercial cosmeceutical messaging can add another source of ambiguity when product claims borrow medical language without corresponding clinical supervision. Cosmeceuticals occupy an intermediate market position between cosmetics and pharmaceuticals and are promoted through the language of tissue repair, molecular renewal, barrier restoration, and regeneration [14]. “Epistemic consumption” describes how scientific vocabulary and expert-coded claims can make product selection appear rational, responsible, and medically informed [14]. For patients already experiencing stigma, appearance monitoring, and limited tolerance for delayed improvement, this language may recast purchasing as self-directed treatment. Because the marketing evidence is not acne-specific, this connection should be interpreted as a plausible persuasion mechanism rather than a demonstrated treatment pathway.

Recent dermatologic commentary has proposed “cosmeticorexia” as a descriptive term for excessive or age-inappropriate cosmetic use, although it is not a formal diagnosis [15]. Direct content evidence shows that the modeled behavior can involve substantial product and ingredient exposure. In an analysis of 100 TikTok videos featuring skincare routines by creators aged 18 years or younger, routines contained a mean of six products, cost an average of $168, and received a mean of 1.1 million views; only 26.2% of daytime routines included sunscreen. Among the 25 most-viewed videos, routines contained a mean of 11 and a maximum of 21 potentially irritating active ingredients, while 76% included at least one potential contact allergen. Experimental engagement by accounts registered as 13-year-old users increased subsequent exposure to similar routine content [16].

These findings extend the concern beyond the accuracy of individual claims: short-form content repeatedly models complex regimens, high active-ingredient exposure, incomplete photoprotection, and continued exposure to similar content after engagement [16]. Because Hales et al. analyzed pediatric skincare videos rather than acne patients, the study demonstrates the routines being modeled, not whether patients receiving acne pharmacotherapy adopt them.

From online recommendations to cutaneous adverse effects and non-adherence

Digital exposure becomes consequential when online recommendations enter treatment behavior. In a cross-sectional survey of 358 adult female acne patients, 88.4% of participants who sought acne information through social media or the internet followed at least one treatment recommendation from those platforms; 20.9% reported willingness to alter physician-prescribed acne treatment based on social media recommendations. Online recommendations therefore entered the treatment sequence as a source of action, including self-medication, home remedies, oral supplements, dietary modification, and over-the-counter product use. In the same cohort, only 0.8% reported significant improvement after following online acne advice, while 12.8% reported worsening [6]. he study demonstrated behavioral uptake of online advice but left unaddressed which specific exposures caused the reported worsening and how patients attributed those symptoms. 

The cutaneous consequences appeared in routine dermatology practice. A national survey of 168 Dutch dermatologists and dermatology residents reported that 91.6% expressed concern about the popularity of multi-step topical cosmetic regimens, and 88.1% regularly encountered skincare-related dermatoses, most commonly irritant eczema, perioral dermatitis, allergic contact dermatitis, and acne exogenica [17]. Frequently implicated product categories included retinoids, exfoliating acids, fragrances, and products marketed as natural. The combination of frequently implicated product categories and reported diagnoses was consistent with cumulative irritant and allergen exposure from complex topical routines [17]. As a clinician survey, it documented complication patterns encountered in practice rather than verified patient-level exposure histories or causation.

Cutaneous adverse effects are associated with non-adherence. In a cross-sectional cohort of 198 patients with dermatologist-confirmed acne vulgaris, non-adherence to anti-acne medication was 50.5% and was significantly associated with treatment-related adverse effects, particularly skin dryness, as well as moderate acne severity and topical therapy [18]. Dryness affected 37.9% of patients with reported side effects, while skin stinging affected 14.1%; non-adherence was significantly higher among patients receiving topical therapy alone and among those experiencing skin dryness or skin stinging [18]. Because social-media exposure and non-prescribed skincare use were not measured in this cohort, this finding established an association between cutaneous adverse effects and non-adherence rather than the origin of those adverse effects. 

Three studies measured adjacent stages rather than one continuous sequence. Ertekin et al. documented uptake of online treatment recommendations [6]; Poelhekken and van der Bent documented dermatoses encountered in association with multi-step cosmetic routines [17]; and Aleissa et al. linked dryness and stinging with medication non-adherence [18]. Together, these studies support a hypothesis-generating model in which digital advice, additional topical exposure, cutaneous symptoms, and reduced treatment persistence are linked as adjacent but separately studied stages; no single study followed the same patients through every stage. The unresolved step was symptom attribution: whether irritation arising during combined prescription and cosmetic use was blamed on the prescribed regimen and prompted premature discontinuation.

Adherence difficulties recur across geographically distinct acne populations, although the reported rates are not directly comparable because the studies used different populations and adherence measures. In the Saudi cohort, non-adherence was 50.5% [18]. Among 2,349 patients across 18 Turkish dermatology centers, 48% reported consistent medication use; leading barriers were forgetfulness, dislike of treatment, and reluctance toward long-term therapy [19]. In Thailand, adherence among 1,237 participants was 33.71%, with cost, adverse effects, lack of noticeable improvement, convenience, and aesthetics influencing persistence or treatment selection [20]. The recurring pattern was not a uniform prevalence estimate but a shared set of treatment frictions: delayed visible benefit, adverse effects, inconvenience, expense, and resistance to prolonged therapy. These are the same points of vulnerability at which transformation-oriented and rapid-result digital messaging may become especially persuasive [6,10-13].

Clinician responses: expectation management, exposure history, and supportive skincare

High physician trust did not eliminate online information-seeking. In a cohort of 100 acne patients, 97% reported that they would trust dermatologist guidance over influencer guidance when the two conflicted, while 67% continued to seek acne information from influencers, most commonly regarding cosmetic products [21]. These findings suggest that distrust is not the sole explanation for digital exposure. In a separate survey of 384 Facebook skincare purchasers in Sri Lanka, source credibility, electronic word-of-mouth credibility, and trust in online communities were positively associated with purchase intention [22]. Together, these studies show that professional trust and online influence can coexist: patients may defer to dermatologists in a stated conflict while continuing to encounter credible-seeming community or commercial information [21,22]. Expressed trust therefore cannot be assumed to eliminate online influence on information seeking and product evaluation.

Because expressed physician trust remains high, the opportunity is to use that credibility during the interval when patients may be evaluating online alternatives. The convergence of treatment-modification, complication, and influencer-use data supports adding a detailed exposure history to acne consultation [6,17,21]. The history should record prescribed agents, over-the-counter acne products, cleansers, exfoliating acids, retinoids, fragrances, supplements, home remedies, and the digital source that prompted each addition. Chronology is essential: when each exposure began, when irritation appeared, which product the patient attributed it to, and whether prescription use changed afterward. This approach may help distinguish persistent acne from treatment irritation, product-induced dermatitis, and incomplete medication use before the original regimen is intensified or abandoned.

Expectation management requires procedural specificity. Reported adherence barriers included limited disease knowledge, confusion about application, weak patient-physician communication, fear or experience of adverse effects, cost, lack of perceived efficacy, regimen complexity, lifestyle burden, forgetfulness, inconvenience, and psychiatric comorbidity [23]. Several of these barriers can be addressed within the consultation. Clinicians can provide a written regimen map identifying which product is therapeutic, which is supportive, how much should be applied, which early effects are expected, which symptoms require stopping therapy, and when clinical response should be assessed. Physical demonstration of topical medication application produced 15% higher mean adherence over six weeks compared with no demonstration, and adherence rose around scheduled follow-up visits, making early follow-up a behavioral intervention rather than a passive monitoring step [23]. Early reassessment was most useful before irritation or lack of visible improvement prompted unsupervised treatment changes [3,23]. When in-person reassessment is impractical, structured digital check-ins could be evaluated as a means of detecting irritation, perceived inefficacy, and newly added topical products before discontinuation.

Supportive skincare should be distinguished from unsupervised product accumulation. Expert consensus recommends acne-appropriate cleansers, moisturizers, and sunscreens to support barrier function, reduce treatment-related irritation, and address post-inflammatory hyperpigmentation when clinically relevant [24]. Selected dermocosmetics may improve tolerability and clinical outcomes when assigned a defined role within medical therapy [24]. This evidence does not support unrestricted over-the-counter experimentation. A minimal cleanser-moisturizer-sunscreen routine may preserve a tangible element of daily self-care while reducing duplicate actives and cumulative irritant exposure; that psychological benefit remains an inference rather than a tested adherence outcome.

These recommendations do not carry equal evidentiary weight. Application demonstration and scheduled follow-up had the closest direct evidence of adherence [23]. Regimen simplification, written instructions, and anticipatory counseling were also supported by established adherence barriers, including confusion about application, adverse effects, poor communication, lack of perceived efficacy, inconvenience, and regimen complexity [23]. In contrast, documenting the digital source of product additions and using structured digital check-ins are proposed digital-era strategies rather than interventions with demonstrated efficacy. These strategies are evidence-informed extensions derived from treatment-modification, complication, and influencer-use data [6,17,21], while dermocosmetic integration is supported mainly by expert consensus [24].

Limitations of the evidence base

The evidence base is limited primarily by cross-sectional design, self-report, and fragmentation across study types. Patient studies measured digital exposure, treatment modification, adverse effects, or medication use at a single time point; content analyses measured available information rather than patient behavior; clinician surveys captured encountered patterns rather than verified exposure histories; and adherence cohorts could not distinguish symptoms caused by prescribed therapy from those caused by non-prescribed products. Several mechanistic and therapeutic arguments also relied on non-acne marketing research, narrative reviews, editorial commentary, and expert consensus. Accordingly, findings from non-acne skincare, consumer-behavior, or general digital-media studies were used only as contextual or mechanistic support and were not treated as direct evidence of behavior, cutaneous outcomes, or adherence among acne patients. Populations and adherence measures were heterogeneous, and the central gap identified above, a single cohort followed from digital exposure through symptom attribution to prescription persistence, has not yet been studied. Additional limitations include restriction to English-language literature, absence of formal duplicate screening or risk-of-bias assessment, and substantial heterogeneity in populations, exposures, outcomes, and study designs, which limits direct comparison across studies. These limitations prevent causal inference but define a specific prospective research agenda. 

## Conclusions

Acne care increasingly occurs in a digital environment where patients may encounter rapid-result product narratives before prescribed therapy has had adequate time to work. The evidence reviewed here supports a plausible, hypothesis-generating model rather than a demonstrated longitudinal sequence. Individual components of the proposed pathway have been observed separately: psychodermatological distress and expectation mismatch are associated with acne burden and treatment-related outcomes; digital content can present product-centered alternatives and model complex routines; and cutaneous adverse effects are associated with poorer medication persistence. However, the longitudinal relationship among these components remains untested. No included study has demonstrated, within the same patients, that digitally influenced product additions produce objective barrier injury, that the resulting symptoms are attributed to prescribed therapy, and that this attribution leads to premature discontinuation.

Dermatologic practice need not wait for the entire pathway to be proven. Dermatologists can define realistic treatment timelines, demonstrate application, simplify regimens, review the chronology of prescribed and non-prescribed exposures, and integrate selected supportive products. These actions address documented adherence barriers while preserving the distinction between interventions with direct adherence evidence and digital-era extensions that still require testing. The objective is not to discourage online acne education or non-prescription skincare, but to prevent unsupervised product accumulation from replacing structured, tolerable treatment. The priority for future research is a prospective acne cohort that measures digital exposure, product additions, barrier change, symptom attribution, and prescription persistence together.
